# VEGFA links self-renewal and metastasis by inducing Sox2 to repress miR-452, driving Slug

**DOI:** 10.1038/onc.2017.4

**Published:** 2017-05-15

**Authors:** M Kim, K Jang, P Miller, M Picon-Ruiz, T M Yeasky, D El-Ashry, J M Slingerland

**Affiliations:** 1 Braman Family Breast Cancer Institute at Sylvester Comprehensive Cancer Center, University of Miami Miller School of Medicine, Miami, FL, USA; 2 Department of Biochemistry and Molecular Biology, University of Miami Miller School of Medicine, Miami, FL, USA; 3 Department of Medicine, University of Miami Miller School of Medicine, Miami, FL, USA

## Abstract

Cancer stem cells (CSC) appear to have increased metastatic potential, but mechanisms underlying this are poorly defined. Here we show that VEGFA induction of Sox2 promotes EMT and tumor metastasis. In breast lines and primary cancer culture, VEGFA rapidly upregulates *SOX2* expression, leading to *SNAI2* induction, EMT, increased invasion and metastasis. We show Sox2 downregulates miR-452, which acts as a novel metastasis suppressor to directly target the *SNAI2* 3′-untranslated region (3′-UTR). VEGFA stimulates Sox2- and Slug-dependent cell invasion. VEGFA increases lung metastasis *in vivo*, and this is abrogated by miR-452 overexpression. Furthermore, *SNAI2* transduction rescues metastasis suppression by miR-452. Thus, in addition to its angiogenic action, VEGFA upregulates Sox2 to drive stem cell expansion, together with miR-452 loss and Slug upregulation, providing a novel mechanism whereby cancer stem cells acquire metastatic potential. Prior work showed EMT transcription factor overexpression upregulates CSC. Present work indicates that stemness and metastasis are a two-way street: Sox2, a major mediator of CSC self-renewal, also governs the metastatic process.

## Introduction

VEGFA is a cytokine that regulates vascular development during embryogenesis and the formation of new blood vessels from pre-existing vascular networks.^1, 2, 3^ VEGFA, secreted by cancer and stromal cells, stimulates endothelial cell invasion and vessel formation.^4^ Without new blood vessel formation, tumor size is restrained due to limited nutrient and oxygen supply. VEGFA is expressed in a variety of tumors and its overexpression is associated with poor prognosis and death from metastasis.^5, 6, 7^ VEGFA functions are not restricted to vasculogenesis and angiogenesis.^8^ Autocrine VEGFA cooperates with EGFR to drive tumor development^9^ and VEGFA has also been shown to drive tumor metastasis.^4, 10, 11^ Indeed, patients with metastatic breast cancer have higher circulating VEGFA levels than those without metastasis.^12^

Bevacizumab, a humanized monoclonal antibody that targets VEGFA, has been applied for the treatment of breast and other malignancies. However, trials in metastatic breast cancer have yielded variable results and the role of this drug is controversial.^13, 14, 15^ Recent work sheds light on the limited results of bevacizumab in most cancers. Hypoxia caused by inhibition of angiogenesis, upregulates *VEGFA* expression, contributing to aggressive disease recurrence.^16, 17^ VEGFA was recently shown to increase tumor-initiating stem cell abundance in skin^18^ and breast cancers,^19, 20^ and in glioblastoma.^21, 22^ The high local VEGFA induced by hypoxia following bevacizumab treatment would thus also promote expansion of the tumor cell subset with the greatest ability to initiate and disseminate tumors.

Cancer stem cells (CSCs) show greater motility and metastatic potential than the bulk tumor cell population and have been postulated to be drivers of tumor metastasis,^23, 24, 25^ but the mechanisms underlying this are not fully characterized. Metastasis requires cell invasion and escape from the primary tumor into the vasculature followed by colonization of secondary sites. Tumor invasion and intravasation are enabled by the epithelial to mesenchymal transition (EMT), a process in which epithelial cells lose polarity and intracellular adhesion, and acquire motility and invasiveness.^26, 27, 28, 29^ The EMT is regulated by diverse molecular networks including TGF-β, Notch, Wnt, Hedgehog and NF-κB signaling pathways, all of which have central roles in cancer invasion and metastasis.^30^ Downregulated expression of the cell adhesion molecule, E-cadherin, is critical for acquisition of the EMT phenotype and tumor invasion.^31^ Many EMT transcription factors repress *CDH1*, the gene encoding E-cadherin, directly or indirectly. Snail,^32, 33^ Slug,^34^ Zeb1^35^ and Zeb2^36^ can bind the *CDH1* promoter and repress its transcription, whereas other factors such as Twist,^37^ Goosecoid^38^ and fork-head box protein C2 (FOXC2)^39^ repress *CDH1* indirectly. Slug, whose expression correlates strongly with loss of E-cadherin, is an important EMT mediator in breast cancer cell models.^40^

The EMT program has been linked to the initiation and/or maintenance of CSCs. Enforced expression of EMT transcription factors has been shown to increase cancer stem cell abundance, and stem-like cells exhibit EMT properties such as increased expression of mesenchymal markers and EMT transcription factors, suggesting a link between cancer stem cells and the EMT process.^41, 42^ However, pathways governing the relationship between cancer stem cells and EMT are not fully defined. VEGFA not only increases the tumor-initiating stem cell population in several different murine and human cancer models,^18, 19, 20, 21, 22^ but is also known to induce EMT and metastasis.^43, 44, 45^ Our prior work showed that VEGFA rapidly activates STAT3 to induce *SOX2* and increase the CSC population in breast and lung models.^19^ Here, we investigated whether upregulation of Sox2 by VEGFA might have a role not only in CSC expansion but also contribute to the activation of EMT and metastasis.

MicroRNAs (miRNAs) are small, noncoding RNAs that regulate transcriptional and post-transcriptional gene expression. Approximately 70% of all genes are regulated by miRNA in eukaryotes.^46, 47^ miRNAs carry out important functions in development, differentiation, cell cycle progression and apoptosis. Mature miRNAs bind complementary sequences in the 3′-untranslated region (3′-UTR) of target genes and repress gene expression by inducing mRNA degradation and/or translational inhibition.^48, 49^ In cancers, miRNA expression is deregulated by amplification, deletion, mutation and epigenetic silencing.^50, 51, 52^ Many miRNAs act as either oncogenes or tumor suppressors to regulate malignant transformation and metastatic progression.^52^ MiRNAs modulate the metastatic process by targeting metastasis suppressor genes or by repressing metastasis promoting genes.^53^ Several miRNAs regulate EMT transcription factors including Zeb1, Zeb2 and Snail.^54, 55, 56^ Indeed, several miRNAs that target EMT transcription factors, such as miR-200 that targets Zeb1^54, 57, 58, 59^ and miR-34 that targets Snail,^60^ also repress cancer stem cell self-renewal.^47^

Here, we identify a novel pathway in which Sox2, a stem cell driver upregulated by VEGFA,^19^ contributes to the activation of EMT. VEGFA leads to induction of the stem cell transcription factor gene *SOX2*. Sox2, in turn, mediates repression of miR-452, which is shown to directly target the 3′-UTR of *SNAI2*, leading to EMT and breast cancer metastasis.

## Results

### VEGFA induces EMT and an increase in motility and invasion in breast models

**Figure 1:**
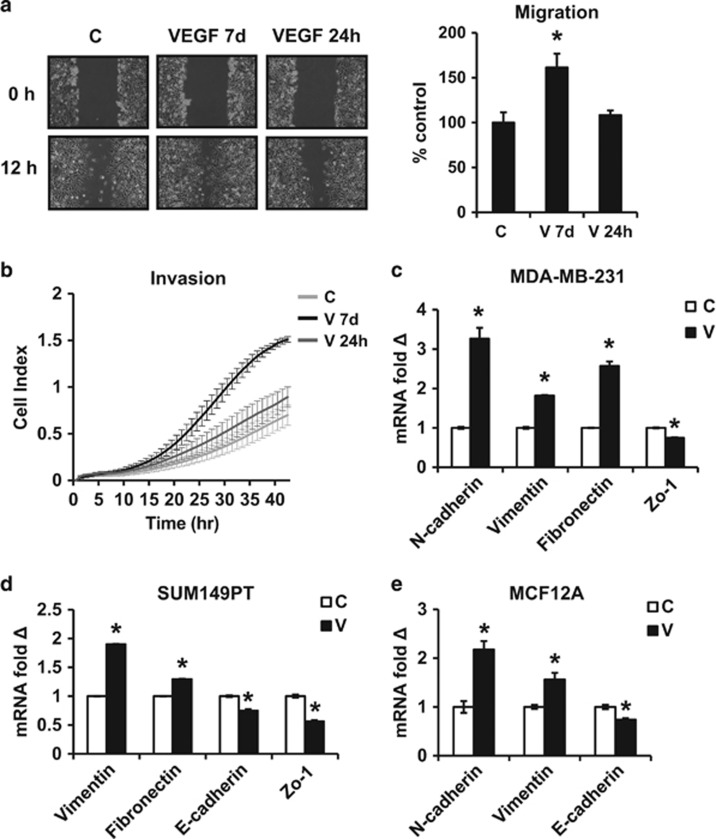
VEGFA induces EMT and an increase in motility and invasion. (**a**) MDA-MB-231 was pre-treated for 24 h or 7 days ±10 ng/ml VEGFA followed by scratch wounding of a confluent monolayer. Photomicrographs were taken at 0 and 12 h and mean migration±s.e.m. graphed versus controls (C). (**b**) MDA-MB-231 pre-treated for 24 h or 7 days±VEGFA were recovered for real-time matrigel invasion assays. Data graphed represent mean +/− s.e.m. for 3 replicates. (**c**–**e**) EMT marker expression was compared by QPCR after 7 days ±10 ng/ml VEGFA (V) in MDA-MB-231 (**c**), SUM149PT (**d**) and MCF12A (**e**). All graphs show mean±s.e.m. Mean values were compared by Student's *t* test. * denotes *P*<0.05 for test versus control.

In addition to its angiogenic effects, VEGFA promotes cancer stem cell expansion.^19^ VEGFA also drives cancer invasion and metastasis in experimental models.^43, 45^ Cancer stem cell expansion is linked to, and potentially driven by, upregulation of EMT transcription factors,^41, 42^ but whether stem cell drivers can also promote EMT has not been fully investigated. To investigate whether VEGFA-mediated CSC expansion might also be linked to EMT activation and metastasis, we tested the effect of VEGFA on motility and invasion in aggressive ER-negative breast cancer models. Since our earlier work showed a prolonged 7-day exposure to VEGFA caused an irreversible increase in stem-like cells,^19^ all experiments used 7 days of VEGFA (10 ng/ml), unless otherwise indicated. VEGFA-treated MDA-MB-231 showed faster migration on wound-healing assays and increased matrigel invasion compared with controls (Figures 1a and b). Results were validated in an ER, PR and Her2 (triple) negative primary breast cancer-derived line, SUM149PT (Supplementary Figure S1a and b).

Acquisition of an EMT phenotype is critical for metastasis. Mesenchymal markers (vimentin, fibronectin and N-cadherin) were upregulated by VEGFA in MDA-MB-231 and SUM149PT lines and in the immortal but not malignantly transformed human mammary epithelial line, MCF12A. Epithelial markers, including one or both of E-cadherin and Zo-1, were decreased in all three cell lines (Figures 1c–e), compatible with a VEGFA-induced EMT.

### VEGFA increases motility and invasion by upregulating Slug in breast cancer cells

**Figure 2:**
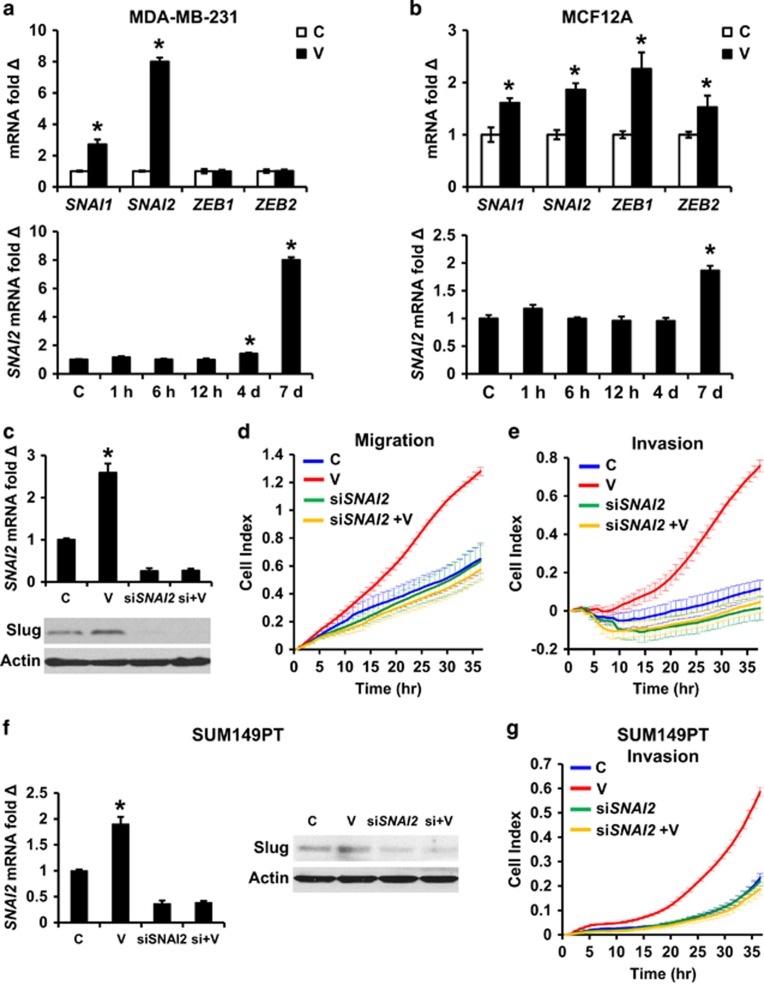
VEGFA increases motility and invasion via slug induction in breast cancer cells. (**a** and **b**) *SNAI1, SNAI2, ZEB1* and *ZEB2* expression levels were assayed by QPCR at indicated times after 10 ng/ml VEGFA in MDA-MB-231 (**a**) and in MCF12A (**b**). (**c**–**e**) The MDA-MB-231 cells were transduced with si*SNAI2* or control siRNA (C) for 48 h. The cells were then treated ±10 ng/ml VEGFA for 7 days and then recovered for assays of *SNAI2* and Slug expression by QPCR and western, respectively (**c**) and assays of migration (**d**) and matrigel invasion (**e**). (**f** and **g**) The SUM149PT cells were transduced with si*SNAI2* or control siRNA for 48 h then treated ±10 ng/ml VEGFA for 7 days followed by assays of *SNAI2* and Slug expression by QPCR and western, respectively (**f**) and by real-time matrigel invasion assay (**g**). All graphs show mean±s.e.m. Mean values were compared by Student's *t* test. * denotes *P*<0.05 for test versus control.

Expression of major EMT-driving transcription factors (EMT-TFs), Slug, Snail, Zeb1 and Zeb2, was induced over a 7-day VEGFA exposure. The temporal patterns of EMT-TF upregulation during prolonged VEGFA exposure for MDA-MB-231 and MCF12A are shown in Supplementary Figures S2A and B. Of these, *SNAI2,* which encodes Slug, was the most strongly induced after 7 days, and was thus investigated further (Figures 2a and b). *SNAI2* knockdown (Figure 2c) prevented VEGFA-mediated increases in cell motility and invasion (Figures 2d and e), indicating VEGFA increases migration and invasion via Slug. VEGFA also upregulated *SNAI2* expression in SUM149PT cells, and *SNAI2* knockdown inhibited VEGF-driven invasion in this second model (Figures 2f and g).

### Sox2 is required for VEGFA-driven Slug upregulation and for increased motility and invasion

**Figure 3:**
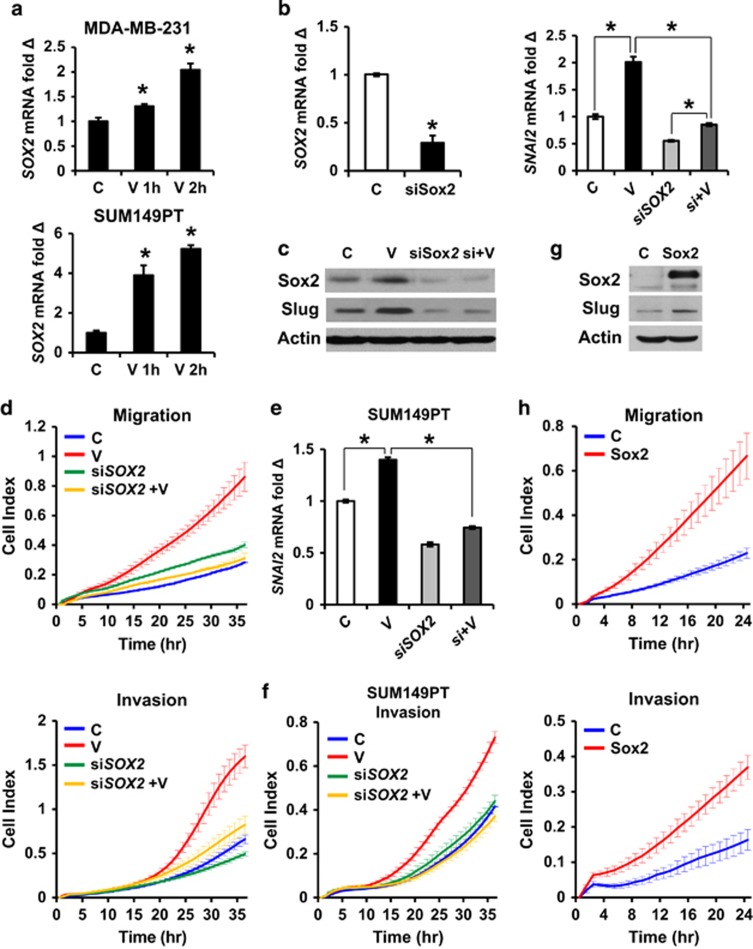
Sox2 is required for VEGFA-driven increases in slug, motility and invasion. (**a**) MDA-MB-231 (top) and SUM149PT (bottom) were treated ±10 ng/ml VEGFA and *SOX2* expression quantitated by QPCR. (**b**) si*SOX2* or control siRNA (C) transfected MDA-MB-231 cells were treated ±10 ng/ml VEGFA for 7 days followed by QPCR for *SNAI2* expression. (**c**) MDA-MB-231 transduced with either si*SOX2* or control siRNA were treated±VEGFA for 7 days before western blot for Sox2 and Slug. (**d**) MDA-MB-231 transduced with either si*SOX2* or control siRNA for 48 h were treated±VEGFA for 7 days followed by assays of migration (top) and matrigel invasion (bottom). (**e** and **f**) SUM149PT cells transduced with si*SOX2* or control siRNA for 48 h were treated±VEGFA for 7 days followed by QPCR for *SNAI2* (**e**) and real-time matrigel invasion assay (**f**). (**g**) MDA-MB-231 cells were transfected with Sox2 or control vector (C), and Sox2 and Slug expression were assayed by western. (**h**) MDA-MB-231 transduced with Sox2 overexpression or control vector and migration (top) and matrigel invasion (bottom) were assayed using Xcelligence. All graphs show mean±s.e.m. Mean values were compared by Student's *t* test. * denotes *P*<0.05 for test versus control or versus indicated condition.

Sox2 drives self-renewal in both embryonic stem cells and in several cancer stem cell models^61, 62^ and is a key mediator of VEGFA-driven CSC expansion.^19^ CSC are thought to be drivers of tumor metastasis and exhibit greater motility and metastatic potential than bulk tumor cells.^23, 24, 63^ Notably, EMT-TF overexpression leads to expansion of cells with stem cell characteristics.^41, 42^ Here we tested whether the reverse is also true and whether the embryonic stem cell factor, Sox2, might mediate VEGFA-driven EMT. Upregulation of *SOX2* expression by VEGFA occurs rapidly, within 1 h in MDA-MB-231 and SUM149PT (Figure 3a) and remains elevated for at least 7 days.^19^ *SOX2* induction precedes that of *SNAI2* by several days. *SNAI2*/Slug upregulation by VEGFA was prevented by *SOX2* knockdown (Figures 3b and c, and Supplementary Figure S3) and Sox2 was also required for VEGFA-mediated increase in cell motility and invasion (Figure 3d) in MDA-MB-231. Findings were validated in the SUM149PT line (Figures 3e and f). Moreover, *SOX2* overexpression was sufficient to increase Slug expression (Figure 3g), invasion and migration in the absence of VEGFA stimulation (Figure 3h). Thus, the rapid VEGFA-STAT3-mediated induction of *SOX2*^19^ not only precedes, but is required for that of *SNAI2* and for the increased migration and invasion following VEGFA exposure in both MDA-MB-231 and SUM149PT.

Although *SOX2* siRNA significantly decreased VEGFA-induced *SNAI2*, *SOX2* siRNA-transduced cells still showed a modest but significant increase of *SNAI2* by VEGFA (Figure 3b right). This may reflect incomplete *SOX2* knockdown by transient siRNA*SOX2* transfection (Figure 3b, left). It is also possible that additional mechanisms govern VEGFA action on Slug.

### miR-452 downregulation is required for VEGFA-mediated increases in Slug and invasion

The *SNAI2* promoter contains a single, putative Sox2 consensus motif, but Sox2 binding to this motif was not detected after VEGFA treatment. Notably, several studies of global Sox2 DNA binding by ChIP-sequencing also failed to show stable binding of Sox2 to the *SNAI2* promoter.^64, 65, 66, 67^ These findings, and our observation that Sox2 is upregulated by VEGFA within hours (Figure 3a), but *SNAI2* expression only increases several days later (Figures 2a and b) suggested that Sox2-mediated *SNAI2* induction is indirect.

**Figure 4:**
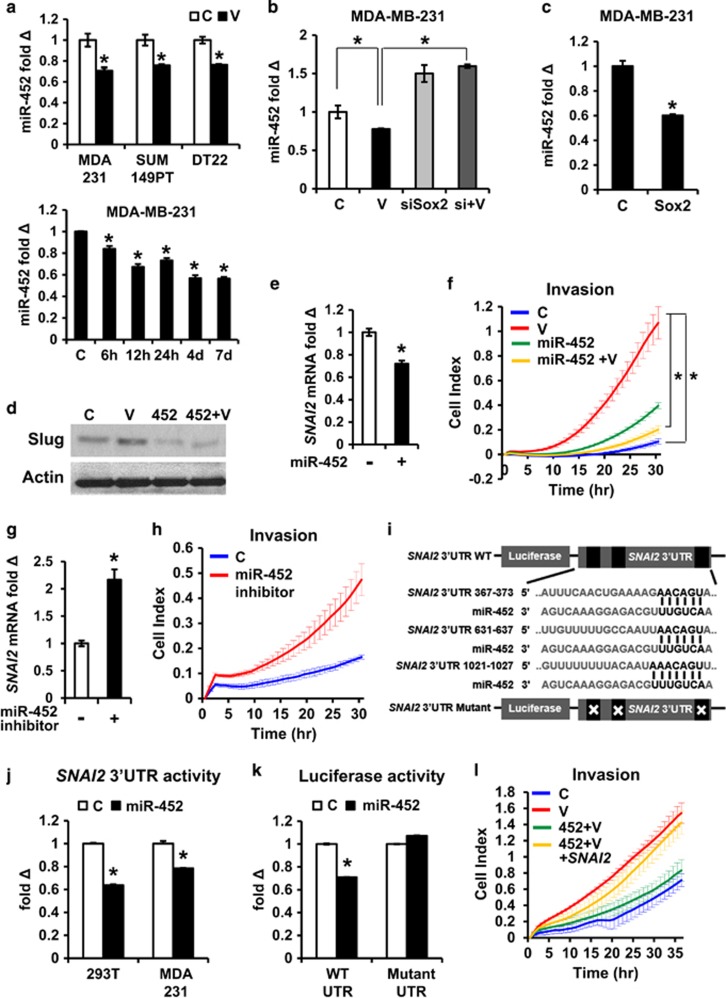
VEGFA- and Sox2-driven miR-452 downregulation mediates Slug upregulation and breast cancer cell invasion, and miR-452 directly targets the *SNAI2* 3′-UTR. (**a**) miR-452 expression (QPCR) was compared±VEGFA for 7 days in MDA-MB-231, SUM149PT and DT22 primary breast cancer culture (top) miR-452 expression levels were assayed by QPCR at indicated times in MDA-MB-231 (bottom). (**b**) The si*SOX2* or control siRNA (C) transduced MDA-MB-231 cells were treated±VEGFA for 7 days and miR-452 expression assayed. (**c**) MDA-MB-231 was transduced with Sox2 or control vector, and miR-452 expression was assayed by QPCR. (**d**–**f**) miRNA control or miR-452 vector transduced cells were treated±VEGFA for 7 days before assays of Slug expression by western (**d**) and *SNAI2* expression by QPCR (**e**) and real-time matrigel invasion (**f**). (**g** and **h**) MDA-MB-231 were transfected with miR-452 antagomir (inhibitor) or antagomir control followed by assays of *SNAI2* by QPCR (**g**) and of invasion as above (**h**). (**i**) Sequence alignment of human miR-452 seed regions with *SNAI2* 3′-UTR. (**j**) 293 T and MDA-MB-231 were transfected with *SNAI2* 3′-UTR luciferase reporter together with miR-452 precursor or control miRNA plasmid and luciferase activity assayed after 48 h. (**k**) *SNAI2* 3′-UTR luciferase reporter plasmid bearing mutations in all three putative miR-452 binding sites show loss of luciferase regulation by transfected miR-452 precursor plasmid after 48 h. (**l**) miR-452 overexpressing MDA-MB-231 was transduced with either human *SNAI2* or control vector, then treated±VEGFA for 7 days followed by real-time matrigel invasion assay. All graphs show mean±s.e.m. Mean values were compared by Student's *t* test. * denotes *P*<0.05 for test versus control or versus indicated condition.

Since Sox2 is known to induce several miRNAs to drive stem cell self-renewal, we investigated whether a miRNA-driven mechanism might govern Slug upregulation. A miRNA screen of MDA-MB-231 cells before and after VEGFA treatment was performed. Among over 700 miRNAs, 47 miRNAs were significantly downregulated by VEGFA (see Supplementary Figure S4). The miRNA target prediction software TargetScan (Version 6.2) was used to identify miRNAs decreased by VEGFA that could potentially target *SNAI2*. Of four potential candidates, miR-452 had the highest probability score for targeting *SNAI2* and was investigated herein. A second miRNA target prediction database (microT-CDS version 5.0) verified miR-452 as a putative regulator of *SNAI2* expression. VEGFA downregulated miR-452 in both MDA-MB-231 and SUM149PT. miR-452 was decreased within 6–12 h and reduced levels persisted after 7 days of VEGFA treatment (Figure 4a, bottom panel). To further validate this finding, DT22, an early passage culture derived from a triple-negative primary human breast cancer was tested. This culture has been extensively validated and its gene expression and tumor marker profiles resemble those of the cancer from which it was derived.^68^ Prolonged exposure of DT22 to VEGFA over 7 days also led to miR-452 loss (Figure 4a, top right). *SOX2* knockdown abrogated the VEGFA-driven loss of miR-452, indicating that Sox2 is required for miR-452 downregulation by VEGFA (Figure 4b). *SOX2* overexpression (Figure 3g) also reduced miR-452 in MDA-MB-231 cells (Figure 4c). To test if downregulation of miR-452 is required for VEGFA to increase invasion, miR-452 was transduced into MDA-MB-231 and stable clones derived (Supplementary Figure S5, top panel). The miR-452 overexpression abrogated VEGFA-driven *SNAI2* upregulation (Figure 4d) and prevented the VEGFA-driven increase in matrigel invasion (Figure 4f). Furthermore, the inhibition of miR-452 by transfection of a miR-452 antagomir increased *SNAI2* expression (Figure 4g) and was sufficient to increase matrigel invasion (Figure 4h). Thus, miR-452 is required for Sox2-driven Slug upregulation and is critical for VEGFA-driven cell motility and invasion.

### miR-452 directly targets the *SNAI2* 3′-UTR to decrease Slug

Stable overexpression of miR-452 decreased *SNAI2* expression (Figure 4e and Supplementary Figure S5, top) and miR-452 antagomir transfection increased *SNAI2* levels in MDA-MB-231 (Figure 4g and Supplementary Figure S5, bottom). miRNAs commonly regulate mRNA expression by binding to the 3′-UTR. There are three putative miR-452 binding sites within the 3′-UTR of *SNAI2* (Figure 4i). To investigate whether miR-452 directly targets the 3′-UTR of *SNAI2* to repress Slug expression, a reporter assay was performed using the 3′-UTR of *SNAI2* to drive luciferase expression. 293T and MDA-MB-231 cells were transfected with a human *SNAI2* 3′-UTR luciferase reporter plasmid together with plasmids encoding either the miR-452 precursor or control miRNA, and luciferase activity was measured after 48 h. miR-452 transfection significantly reduced luciferase activity, indicating miR-452 targets the *SNAI2* 3′-UTR to repress Slug expression (Figure 4j).

A mutant *SNAI2* 3′-UTR luciferase vector was constructed in which all three putative miR-452 binding sites were mutationally disrupted. When this mutated vector was co-transfected into 293T cells with the miR-452 precursor plasmid, luciferase activity was not impaired. Thus one or more of these sites is required for miR-452 to inhibit *SNAI2* expression (Figure 4k).

To test whether *SNAI2* overexpression could rescue the inhibitory effect of miR-452 on VEGFA-induced invasion, miR-452 overexpressing MDA-MB-231 cells were transduced with either control vector or human *SNAI2* cDNA lacking the 3′-UTR region. As noted above, miR-452 overexpression abrogated the increased invasion by VEGFA (Figure 4f). Overexpression of this 3′-UTR-deficient *SNAI2* vector rescued the inhibitory effect of miR-452 on cell invasion (Figure 4l), consistent with the notion that miR-452 targets *SNAI2*. Thus, VEGFA-mediated miR-452 downregulation is critical for the induction of *SNAI2* and for Slug action on cell motility and invasion.

### Repression of miR-452 is required for VEGFA-dependent cancer metastasis *in vivo*

**Figure 5:**
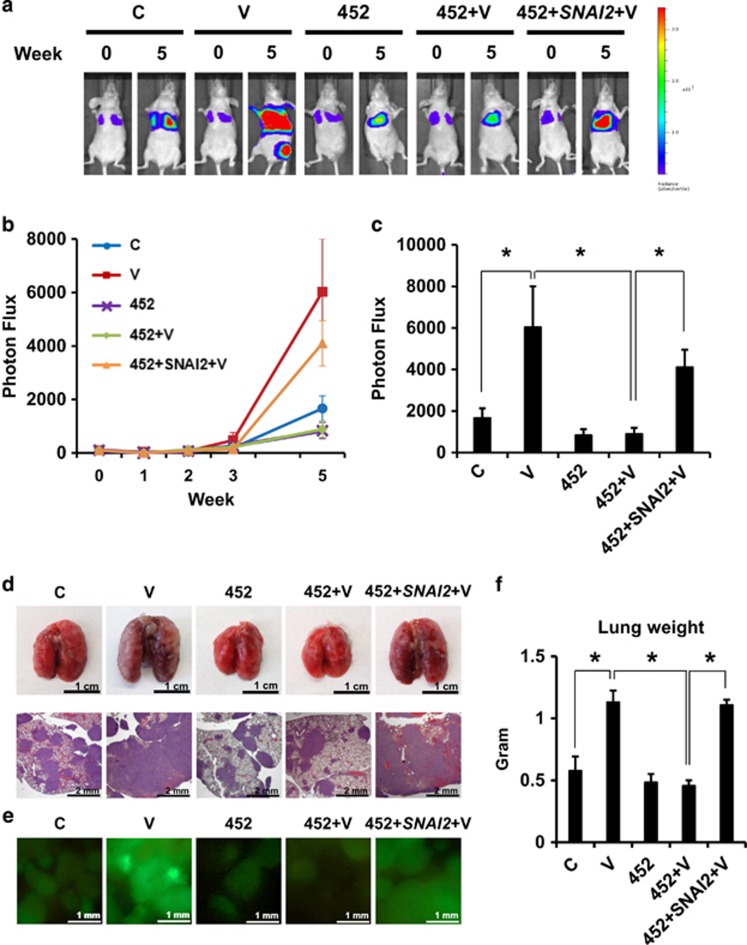
miR-452 repression is required for VEGFA-induced cancer metastasis *in vivo.* (**a**) MDA-MB-231-luc expressing the indicated vectors were pre-treated ±VEGFA and injected via tail vein into nude mice as described in the ‘Materials and methods’ section. Representative bioluminescence images of tumor bearing mice at 0 and 5 weeks are shown. The color scale depicts photon flux (photons/s) from xenografted mice. (**b**) Mean bioluminescence/time of lung metastasis in xenografted mice, graphed as normalized photon flux/time. (**c**) Mean bioluminescence at 5 weeks (**d**) Representative images and photomicrographs of lung tumors from indicated groups. (**e**) Representative immunofluorescence images (× 4) of GFP-positive metastasis observed immediately *ex vivo*. (**f**) Mean lung weights. All graphs show mean±s.e.m. Mean values were compared by Student's *t* test. * denotes *P*<0.05 for test versus control or versus indicated condition.

Although VEGFA has been shown to drive cancer metastasis,^45, 69^ mechanisms thereof are largely unknown. To test whether VEGFA drives metastasis *in vivo* through regulation of miR-452 and Slug, MDA-MB-231 cells were pre-treated with VEGFA for 1 week before injection by tail vein into nude mice, without further VEGFA treatment after tumor cell injection. The animals were monitored by *in vivo* imaging system. VEGFA pre-treated cells gave rise to a significant increase in lung tumor establishment as measured by tumor bioluminescence on *in vivo* imaging system over the next 5 weeks compared with mock-treated cells (Figures 5a–d). VEFG also increased green fluorescence of lung tumors (Figures 5e). miR-452 overexpressing cells failed to respond to VEGFA, and showed no effect of VEGFA pre-treatment on tumor metastasis. Notably, transduction of a *SNAI2* cDNA vector lacking the 3′-UTR into miR-452 overexpressing cells overcame the effect of miR-452 to inhibit VEGFA-stimulated metastasis *in vivo* (Figures 5a–e). VEGFA treatment does not affect MDA-MB-231 cell cycle progression or population growth.^19^ Overexpression of miR-452 and *SNAI2* did not change cell proliferation (Supplementary Figure S6), thus differences in the metastatic tumor burdens of each group are not due to differences in growth rates.

The animals injected with VEGFA pre-treated cells showed extensive areas of confluent tumor growth in the lungs on microscopic analysis, precluding accurate enumeration of tumor nodules (Figures 5d). As a second measure of lung tumor burden, lung weights were measured. The lung weights were significantly increased in animals injected with VEGFA-pretreated cells, whereas those of mice injected with VEGFA-treated miR-452 overexpressing cells were not increased compared with controls. Finally, *SNAI2* transduction into the miR-452 overexpressing cells yielded similar lung weights to those in the VEGFA-treated group (Figure 5f). Thus miR-452 repression is required not only for VEGFA-dependent Slug upregulation *in vitro*, but also for increased cancer metastasis *in vivo*.

### *VEGFA*, *SOX2*, *SNAI2*, *miR-452* and *GABRE* expression and prognosis in primary breast cancers

**Figure 6:**
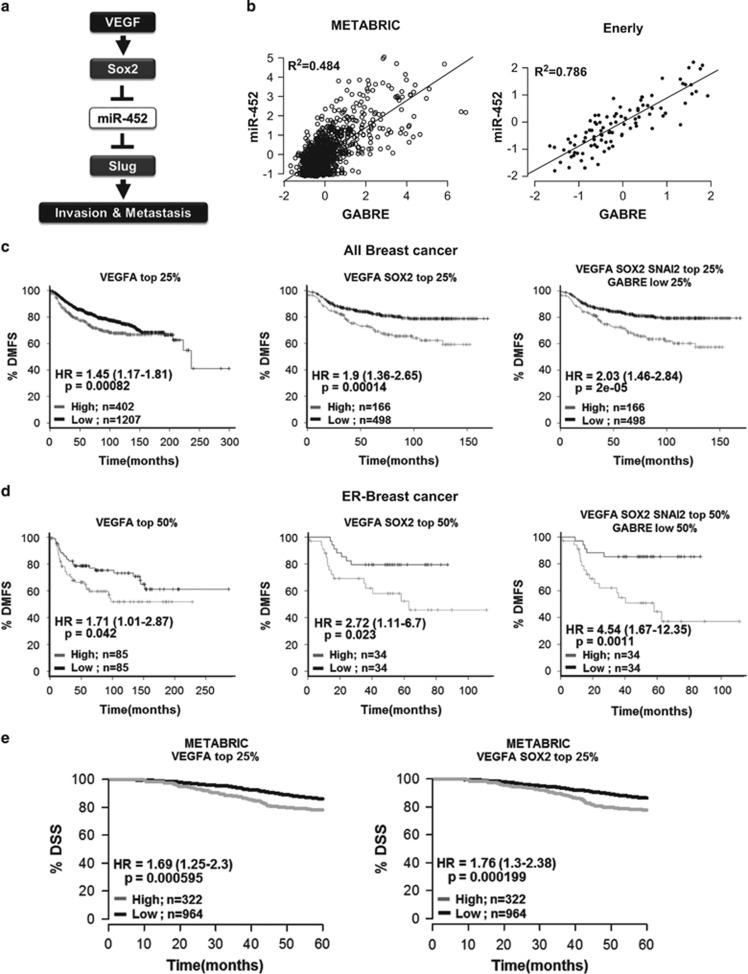
Prognostic value of VEGFA, SOX2, SNAI2, miR-452 and GABRE expression in breast cancer patients. (**a**) Model of mechanism by which VEGFA increases breast cancer invasion and metastasis through Sox2, miR-452 and Slug. (**b**) Correlation of miR-452 and *GABRE* expression in the METABRIC (left) and Enerly (right) primary breast cancer data sets; *R*^2^ values for Pearson's correlation are indicated. (**c** and **d**) Kaplan–Meier plots of distant metastasis-free survival (DMFS) of all breast cancer patients (**c**) and those with ER-negative breast cancer (**d**), stratified by *VEGFA* expression alone or with sequential inclusion of downstream pathway genes (*SOX2*, *GABRE*, *SNAI2*); data were analyzed and plots generated using the KM Plotter web tool (http://kmplot.com/analysis/). Patients with available clinical data: DMFS, *n*=1609. (**e**) Kaplan–Meier survival plot of disease-specific survival (DSS) of breast cancer patients from the METABRIC data set classified by top quartile *VEGFA* expression (left) or the top 25% patients with the highest mean expression of both *VEGFA* and *SOX2* were classified as ‘high’ (right). Hazard ratios, HR (95% CI), were determined using Univariate Cox Proportional Hazards analysis and the *P*-values from the log-rank test are shown in each graph.

Our *in vitro* and *in vivo* models suggest a mechanism in which VEGFA induces EMT and metastasis by activating Sox2, resulting in de-repression of *SNAI2* through loss of miR-452 (Figure 6a). To validate our findings *in vivo*, we tested whether high *VEGFA* alone or together with high *SOX2, SNAI2* and decreased miR-452 expression might identify prognostic subsets of primary human breast cancers. miR-452 is expressed as an intronic transcript from the *GABRE* gene locus.^70^ Pearson’s correlation analysis of two independent breast cancer data sets, the METABRIC and Enerly data sets,^71, 72^ respectively, showed that miR-452 expression correlated strongly with that of its parent transcript, *GABRE* (*R*^2^ values of 0.484 and 0.786 in the METABRIC and Enerly data sets, respectively; Figure 6b), indicating *GABRE* can be used as a surrogate for miR-452 expression in data sets, such as KM Plotter, that lack microRNA data.

As Sox2 regulates *SNAI2* expression via miR-452, we next tested whether high *VEGFA* expression (top quartile) alone or in combination with high *SOX2*, high *SNAI2* and the lowest quartile *GABRE*/miR-452 expression was associated with distant metastasis-free breast cancer survival (DMFS) in the KM Plotter data set. Differences between groups are shown by graphed Kaplan–Meier curves and hazard ratios (HRs) from Univariate Cox Proportional Hazards analysis. Log-rank comparison of outcome curves was done and *P*-values are presented in each graph. Of 1609 primary breast cancers in the KM Plotter data set, those with high *VEGFA* expression alone showed significantly poorer DMFS (*n*=1609, HR (95% confidence interval [CI])=1.45 [1.17–1.81] *P*=0.00082; Figure 6c). We next tested elevated *VEGFA* and *SOX2* expression, and then evaluated tumors with high levels of *VEGFA, SOX2* and *SNAI2,* and low *GABRE* (a surrogate for miR-452) expression. Tumors in the top quartile of both *VEGFA* and *SOX2* expression showed significantly worse DMFS (HR for recurrence [95% CI]=1.9 [1.36–2.65] *P*=0.00014; Figure 6c), while those with high expression of *VEGFA, SOX2* and *SNAI2* and the lowest quartile *GABRE* expression showed an even greater risk of relapse (HR [95% CI]=2.03 [1.46–2.84] *P*=2.0e−05; Figure 6c).

A similar analysis showed *VEGFA* expression was of greater prognostic importance in breast cancers defined as ER-negative by clinical ER protein immunohistochemistry (*n*=170 with both VEGFA and ER data). Although the top quartile *VEGFA* expression associated with shorter DMFS (HR [95% CI]=2.87 [1.69–4.86] *P*= 4.2e−05; Supplementary Figure S7), median cutoffs were used to classify ‘high’ or ‘low’ expressers due to the reduced sample size. Using median cutoffs, high *VEGFA* associated with shorter DMFS (HR [95% CI]=1.71 [1.01–2.87] *P*=0.042; Figure 6d). ER-negative cancers with high *VEGFA* and *SOX2* expression showed significantly shorter DMFS (HR=2.72 [95% CI, 1.11–6.7] *P*=0.023; Figure 6d). Remarkably, among ER-negative breast cancers, those with high *VEGFA, SOX2* and *SNAI2* together with low *GABRE* expression showed a 4.54-fold higher risk of metastasis, with DMFS (HR [95% CI]=4.54 [1.67–12.35] *P*=0.0011, Figure 6d). These KM Plotter data identify a very aggressive population within all cancers and in ER-negative breast cancers, in which the mechanistic pathway identified herein appears to be activated.

To validate these findings in an independent patient group, a similar analysis was carried out for disease-specific survival (DSS) in the METABRIC breast cancer data set (*n*=1286). Analysis of this second independent patient cohort confirmed the prognostic significance of *VEGFA* and showed that elevation of both *VEGFA* and *SOX2* expression associated with a worse survival than did *VEGFA* elevation alone. High *VEGFA* alone conferred a 1.69-fold higher risk of death (DSS HR (95% CI) =1.69 (1.25–2.3), *P*=0.000595: Figure 6e, left), and METABRIC cancers in the top quartile of both *VEGFA* and *SOX2* had even worse outcome (DSS HR (95% CI) =1.76 (1.3–2.38), *P*=0.000199; Figure 6e, right).

## Discussion

VEGFA is best known as an angiogenic agent,^73^ but it also promotes cancer invasion and metastasis through mechanisms that are not fully understood. VEGFA not only creates a vascular niche for expanding stem cells,^21^ it was recently shown to increase the stem-like cell population in certain human malignancies, including breast cancer.^18, 19, 20, 22^ Hypoxia, caused by angiogenesis inhibitors, stimulates *VEGFA* gene expression, and would thus contribute to CSC expansion^74^ and disease recurrence and progression.^16, 17^

CSC have been implicated as drivers of tumor metastasis, however, the molecular pathways linking stemness and induction of metastasis are not fully elucidated. Populations bearing surface CSC markers^75, 76, 77^ or that are enriched for ALDH1 activity^78^ have been shown to have greater motility, invasiveness and metastatic potential than the bulk of the cancer population. Recent work in a pancreatic model showed EMT and dissemination may precede overt tumor invasion.^79^ Circulating tumor cells could be detected during *in situ* tumor growth before overt invasion. Circulating tumor cells bearing the CSC marker, CD44+, showed much more aggressive self-renewal and tumor-generating potential than CD44+-positive cells from the primary tumor site, indicating that escape of stem-like cells from the primary tumor environment is linked to increased self-renewal potential.^79^

Sox2 is an important mediator of self-renewal in embryonic stem cells and is an oncogenic driver of CSC in several cancer models, including breast cancer.^19, 25, 61, 62^ Our prior work showed that VEGFA mediates CSC expansion via STAT3-driven *SOX2* induction in breast and lung cancer models.^19^ Proinflammatory cytokines that are upregulated on breast cancer cell invasion into fat also induce *SOX2* to drive CSC self-renewal.^25^ *SOX2* knockdown can decrease both CSC and experimental lung metastasis^62^ and *SOX2* expression is associated with colon cancer metastasis.^80^ Present work reveals Sox2 is necessary for VEGFA-driven *SNAI2* induction, EMT and invasion of breast cancer cells and provides a mechanistic link between VEGFA-stimulated CSC expansion via *SOX2* induction,^19^ and the upregulation of metastatic potential.

EMT arising from overexpression of various EMT-TFs has been shown to increase tumor-initiating cell abundance;^41, 42^ moreover, stem-like cells exhibit EMT properties such as increased mesenchymal markers and EMT transcription factor expression^81^ suggesting an intimate relationship between CSCs and EMT. Mammary cell lines overexpressing various EMT-TFs showed PLCγ-mediated PKC activation leading to a c-Jun/Fra1-induced CSC transcriptional program.^82^ TGF-β and TNFα pathways interact to drive both EMT and upregulate breast CSC properties.^83^ Elegant *in vitro* and *in vivo* studies in a Trp53-null mouse breast cancer model showed cross-talk between transformed mesenchymal cells and tumor-initiating subpopulations. The mesenchymal cells produced stimulatory ligands driving CSC surface receptors to increase tumorigenicity and metastasis via both Wnt/Fzd7 and CXCL12/CXCR4 pathways,^84^ suggesting that heterogeneous cell populations with differing stem cell self-renewal may interact with each other to drive pathways governing both self-renewal and metastasis.

miRNAs regulate many processes central to oncogenesis.^47^ Several miRNAs oppose EMT by targeting EMT transcription factors.^85^ miR-200 targets Zeb1 and Zeb2^54,57–59^ as does miR-138,^86^ and Snail is targeted by miR-30a^56^ and miR-34.^60^ A number of miRNAs not only regulate EMT but also serve as key CSC regulators. For example, miR-200 not only inhibits EMT by suppressing Zeb1/2, but also downregulates stem-like cells by targeting Bmi1^87, 88^ and the Notch pathway.^89^ In addition to its action on Snail, miR-34a also decreases CSC by targeting Myc^90^ and downregulates CD44 expression to decrease prostate CSC.^91^ miR-128-2 targets both the EMT mediator Snail, and CSC drivers Nanog, KLF4^92^ and Bmi1.^93^

Here we identify miR-452 at the interface between VEGFA-activated CSC self-renewal and EMT, providing a novel connection between VEGFA, induction of the embryonic stem cell transcription factor, Sox2 and EMT. Sox2 not only governs CSC expansion, but also mediates acquisition of EMT and metastatic potential. VEGFA-induced *SOX2* expression is required not only for CSC expansion,^19^ but also for VEGFA-mediated *SNAI2* induction. VEGFA increased *SOX2* expression within an hour, but EMT-TF levels, and in particular that of Slug, rose over several days, suggesting that Sox2 affects Slug indirectly, via an intermediary mechanism. Our miRNA screen identified miR-452 as a putative metastasis suppressor, significantly downregulated by VEGFA in MDA-MB-231 cells. miR-452 is downregulated in breast cancers compared with normal breast tissue.^94^ We show miR-452 targets *SNAI2* directly. MiR-452 overexpression abrogated the VEGFA-mediated upregulation of *SNAI2* expression and cell invasion, and miR-452 antagomir was sufficient to upregulate *SNAI2* expression and cell invasion. Sox2 upregulation by VEGFA mediates the loss of miR-452, and miR-452 loss is required for Slug upregulation and for VEGFA-driven cell motility, invasion and metastasis in breast cancer models. Notably, miR-452 expression correlates inversely with glioblastoma survival and inhibits glioma stem cells and tumorigenesis by targeting CSC mediators, Bmi1, LEF1 and TCF4.^95^ Thus, miR-452 may not only regulate Sox2-driven EMT via Slug, but may also serve a dual role at the interface between EMT and CSC regulation.

The pathway linking VEGFA to Sox2 upregulation, miR-452 loss and *SNAI2* induction is supported by our analysis of two major data sets including over 2500 primary human breast cancers. Although high intratumor VEGFA levels detected by immunohistochemistry have been linked to poor breast cancer outcome, most studies have been small and results controversial.^96, 97^ Our analysis showed breast cancers in the highest quartile of VEGFA expression fare worse than all others, and the prognostic value of high VEGFA levels is increased by sequential addition of high *SOX2, SNAI2* and decreased *GABRE* (a surrogate for miR-452) expression. This finding is less important for its prognostic significance than it is as a confirmation of the molecular pathway identified herein. Among aggressive breast cancers expressing high VEGFA, those with SOX2 overexpression define an even more aggressive subgroup in the two independent data sets evaluated.

VEGFA is a critical mediator of tumor progression. It acts to generate a vascular niche for CSC through autocrine and paracrine action on both tumor and microenvironmental components, and links CSC self-renewal to the acquisition of metastatic potential. To date, targeting VEGFA has had limited success in cancer, and this may be due to anti-angiogenics causing tumor hypoxia, leading to upregulation of both VEGFA and CSC. Since treatment of metastasis is the final therapeutic frontier, it is hoped that mechanistic insights linking VEGFA to tumor initiation and the acquisition of metastatic potential will ultimately generate new strategies for VEGF pathway-targeted intervention.

## Materials and methods

### Cell lines and reagents

Luciferase-tagged MDA-MB-231 (from J Massague, MSKCC, New York, NY, USA), Lenti-X 293 T cells (from Clontech, Mountain View, CA, USA) and MCF12A line were cultured as described.^19^ Both lines were verified by STR profiling. SUM149PT cells were provided by S Ethier (Medical University of South Carolina, Charleston, SC, USA) and cultured in Ham's F12 medium with 5% FBS, 5 μg/ml insulin, 1 μg/ml hydrocortisone and 1 mm HEPES.^98^ All lines were mycoplasma free.

### Scratch assay

MDA-MB-231 cells were seeded into six-well plates, grown to confluence and wound-healing scratch assays were performed as in Larrea *et al.*,^99^ and the cells were photomicrographed after 12 h using an Olympus CKX41 microscope.

### Transwell migration and invasion assays

Real-time cell analysis from Xcelligence (ACEA Biosciences, San Diego, CA, USA) was used for automated transwell migration and invasion from serum-free toward serum-containing medium was as described^100^ and plotted as mean cell index±s.e.m. for at least three independent wells per group.

### Quantitative real-time PCR and miRNA RT–PCR

QPCR was performed at least thrice and mean Ct values normalized to GAPDH or 18S values. mRNA isolation used miRNeasy mini kit (Qiagen, Hilden, Germany) and cDNA synthesys used NcodemiR First-Strand cDNA synthesis kit (Invitrogen, Carlsbad, CA, USA). miR-452 levels were assayed by QPCR. PCR primers for EMT markers and transcription factors assayed, and for miR-452 are in Supplementary Figure S8.

### miRNA screen and antagomiR

MDA-MB-231 cells were treated ±10 ng/ml VEGFA for 7 days followed by Ready-to-Use PCR microRNA array, Human panel I+II in 384-well plates from Exiqon (Woburn, MA, USA). miRCURY LNA miR-452 antagomir and miRCURY LNA miRNA antagomir control were purchased from Exiqon (Woburn, MA, USA) and transduced per manufacturer.

### siRNA analysis and western blots

SiRNA pools of three to five target-specific 19–25 nucleotide siRNAs designed to knockdown Slug/Sox2 and control siRNAs were purchased from Santa Cruz Biotechnology (Dallas, TX, USA) and used per manufacturer. Western blots were as described^19^ using antibodies: anti-Slug (#9585) and Sox2 (#3579) from Cell Signaling (Denver, CO, USA); β-actin (#A1978) from Sigma-Aldrich (St Louis, MO, USA).

### Lentivirus production and transduction

Human *SNAI2* (EX-T1290-Lv155), control vector (EX-NEG-Lv155), hsa-mir-452 (HmiR0407-MR03) and miRNA scrambled control clone (CmiR0001-MR03) lentivirus vectors were purchased from GeneCopoeia (Rockville, MD, USA). Lentivirus vectors were co-transfected with Delta VPR and CMV VSVG plasmids (Addgene, Cambridge, MA, USA) into Lenti-X 293T cells with Lipofectamine Plus. Viral supernatants at 48 h were concentrated by ultracentrifugation for 2 h at 22 000 r.p.m. at 4 °C. MDA-MB-231 was infected with virus in polybrene (10 μg/ml) as described.^19^ Stable expression was confirmed by GFP fluorescence visualization and western.

### Luciferase assays

293 T and MDA-MB-231 were transfected with human *SNAI2* 3′-UTR luciferase reporter (GeneCopoeia, Rockville MD, USA) plasmid together with miR-452 or control miRNA plasmid. After 48 h, Firefly and Renilla luciferase reporter activity luciferase activity was measured using Luc-Pair Duo-Luciferase Assay Kit 2.0 (GeneCopoeia, Rockville MD, USA) per manufacturer's instructions.

### Experimental lung metastasis assay

MDA-MB-231-luc and controls transduced with miR-452 or miR-452+*SNAI2* were pre-treated ±VEGFA for 7 days, before injection of 5 × 10^5^cells via tail vein into 4–6-week female Balb/C nude mice as described.^19^ Each experimental group contained 10 animals. The mice were imaged by *in vivo* imaging system (Xenogen, Caliper, Hopkinton, MA, USA) and bioluminescence (photon flux) was quantified with time as described.^100^ All animal work was carried out in compliance with the Institutional Animal Care and Use Committee in the University of Miami.

### Statistical analysis and expression analysis of *VEGFA, SOX2, SNAI2, GABRE* genes and miR-452

The METABRIC data set contains gene expression data for 2136 and microRNA expression for 1448 primary breast cancer samples, with both available in 1302 samples, together with clinical information and DSS outcome data.^71, 101^ METABRIC and the independent Enerly primary breast cancer data set, containing 101 cases,^72^ were used to identify a correlation between miR-452 and GABRE expression by Pearson’s correlation. The KM plotter data set contains gene expression from primary human breast cancers (*n*=2553) and was used for the analysis of distant metastasis-free survival (DMFS).

For clinical outcome analysis, expression quartiles were used to test whether VEGFA expression alone, or with the other genes, associated with poor DSS (METABRIC) or with poor DMFS (KM plotter) using Kaplan–Meier analysis and Univariate Cox proportional hazards analysis identified hazard ratios with 95% CI. DSS or DMFS curves were also compared using the log-rank test and the *P*-value from this analysis was displayed in each graph. Data analysis was performed using R statistical software or by using the KM plotter web tool, as in Gyorffy *et al.*^102^ Mihaly *et al.*^103^

For *in vitro* work, data are graphed from ⩾3 biologic experiments as means±s.e.m. Means were compared with two-tailed Student’s *t*-tests. *P*-values <0.05 were considered statistically significant. Statistical differences of a real-time cell analysis data between invasion rates used the ‘Compare Growth Curves’ function (http://bioinf.wehi.edu.au/software/compareCurves/).
