# Supplementary material for: VEGFA links self-renewal and metastasis by inducing Sox2 to repress miR-452, driving Slug
Source: Oncogene. 2017 May 15;36(36):5199–211. doi: 10.1038/onc.2017.4 (PMC5596211; doi:10.1038/onc.2017.4)
Supplement: Supplementary Information [file onc20174x1.ppt]

## Slide 1
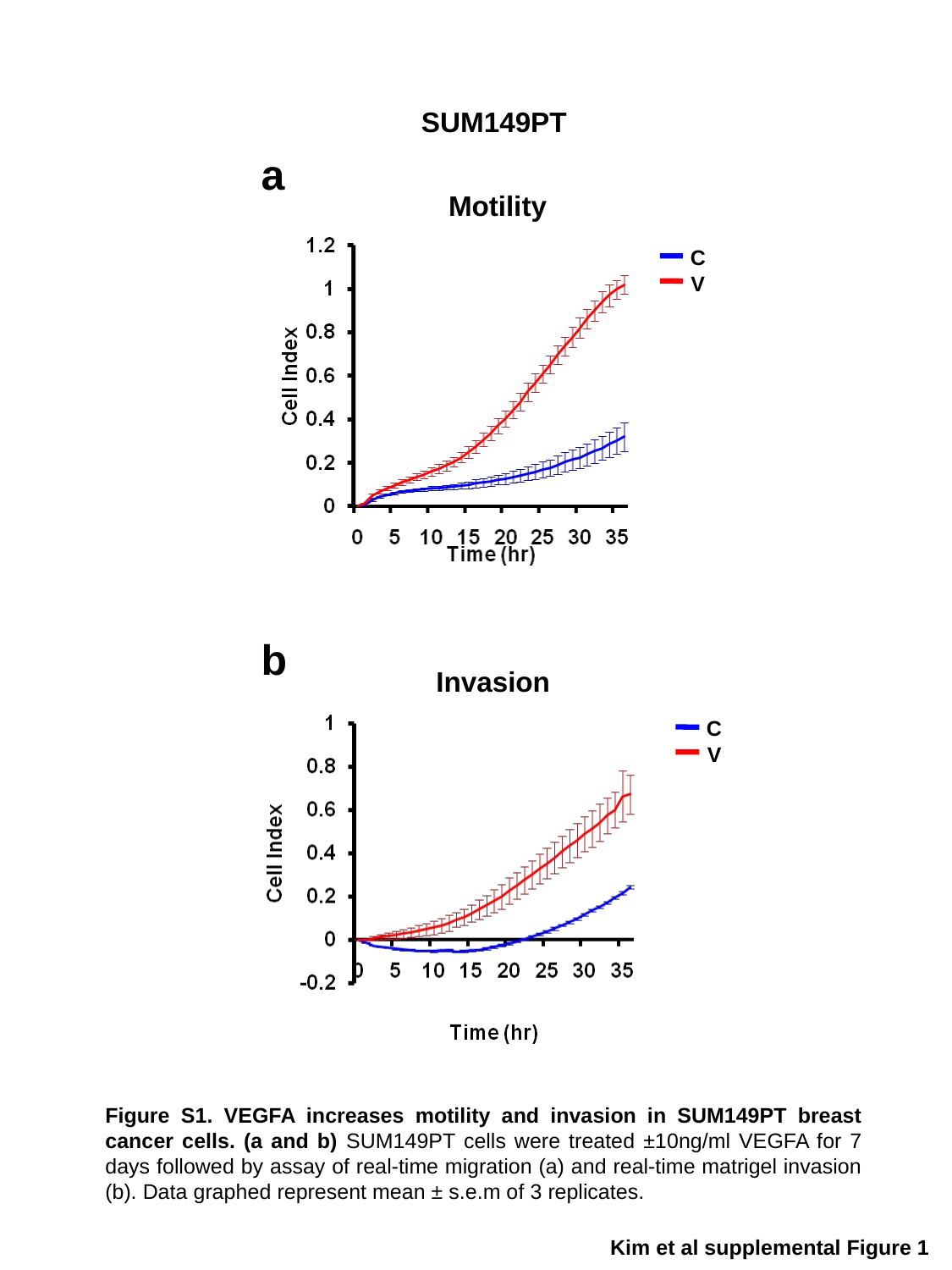

SUM149PT
a
Motility
C
V
b
Invasion
C
V
Figure S1. VEGFA increases motility and invasion in SUM149PT breast cancer cells. (a and b) SUM149PT cells were treated ±10ng/ml VEGFA for 7 days followed by assay of real-time migration (a) and real-time matrigel invasion (b). Data graphed represent mean ± s.e.m of 3 replicates.
Kim et al supplemental Figure 1

## Slide 2
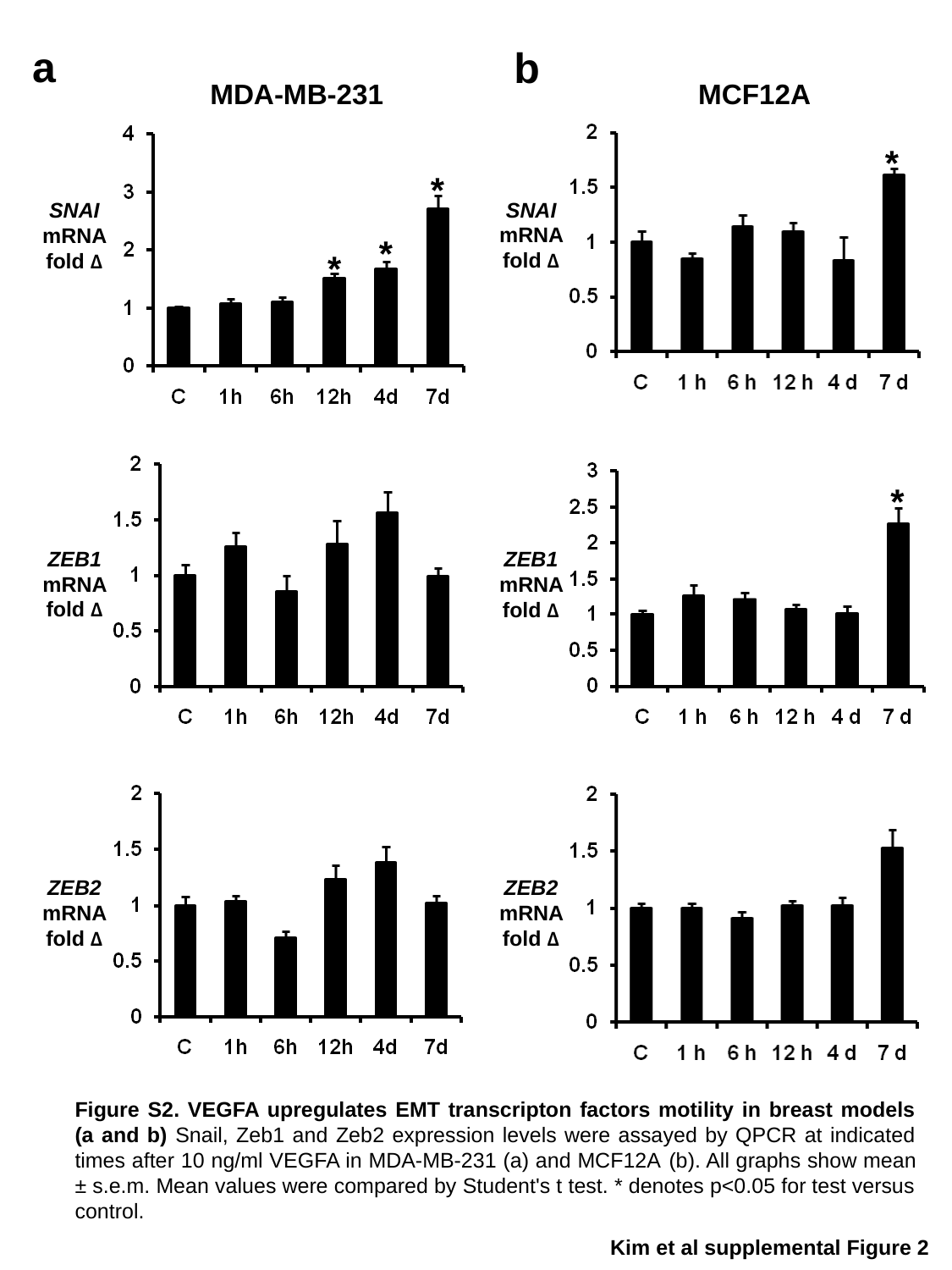

a
b
MDA-MB-231
MCF12A
*
SNAI
mRNA fold Δ
*
SNAI
mRNA fold Δ
*
*
ZEB1
mRNA fold Δ
*
ZEB1 mRNA fold Δ
ZEB2
mRNA fold Δ
*
ZEB2
mRNA fold Δ
Figure S2. VEGFA upregulates EMT transcripton factors motility in breast models (a and b) Snail, Zeb1 and Zeb2 expression levels were assayed by QPCR at indicated times after 10 ng/ml VEGFA in MDA-MB-231 (a) and MCF12A (b). All graphs show mean ± s.e.m. Mean values were compared by Student's t test. * denotes p<0.05 for test versus control.
Kim et al supplemental Figure 2

## Slide 3
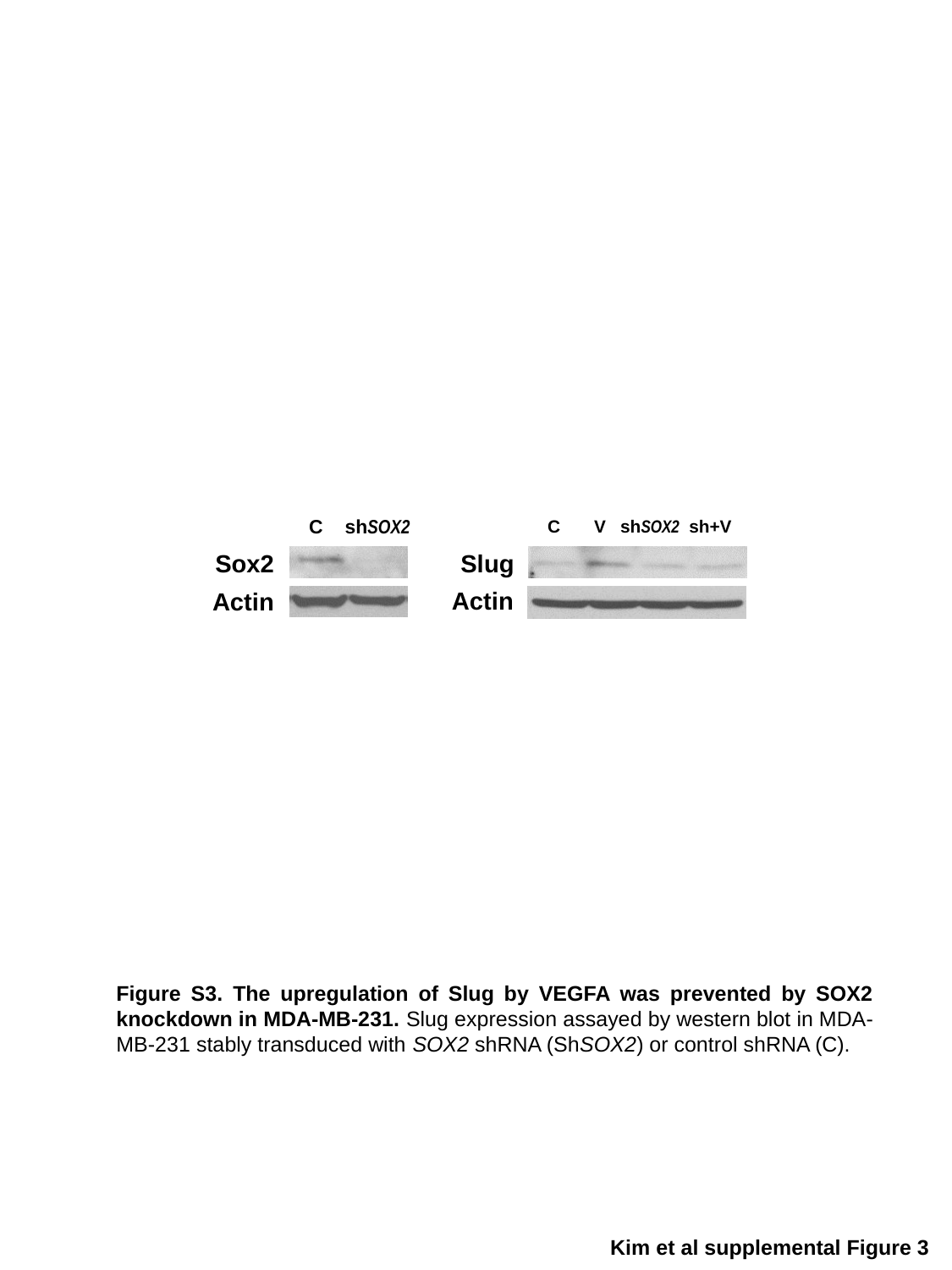

C shSOX2
Sox2
Actin
C V shSOX2 sh+V
Slug
Actin
Figure S3. The upregulation of Slug by VEGFA was prevented by SOX2 knockdown in MDA-MB-231. Slug expression assayed by western blot in MDA-MB-231 stably transduced with SOX2 shRNA (ShSOX2) or control shRNA (C).
Kim et al supplemental Figure 3

## Slide 4
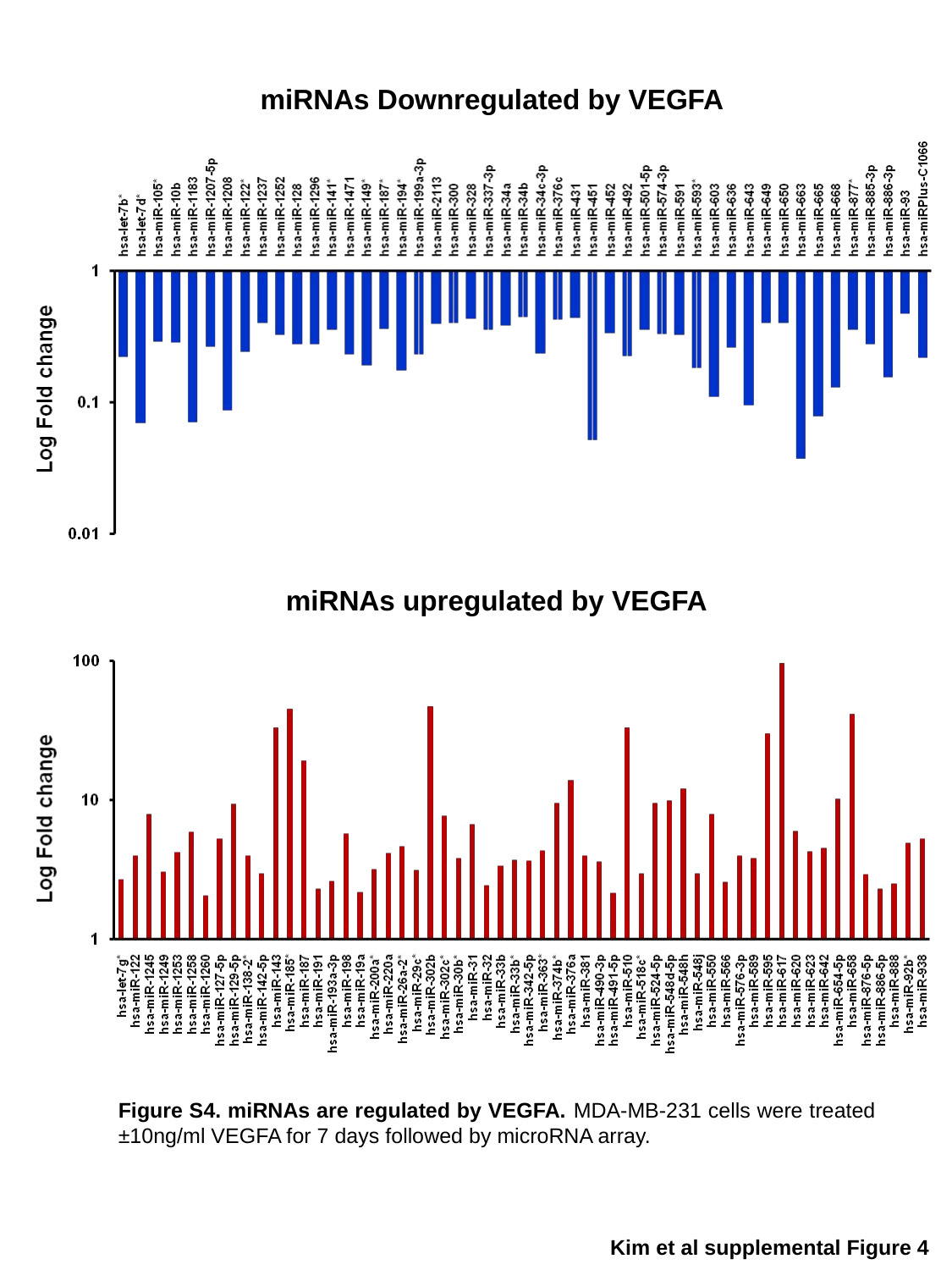

miRNAs Downregulated by VEGFA
miRNAs upregulated by VEGFA
Figure S4. miRNAs are regulated by VEGFA. MDA-MB-231 cells were treated ±10ng/ml VEGFA for 7 days followed by microRNA array.
Kim et al supplemental Figure 4

## Slide 5
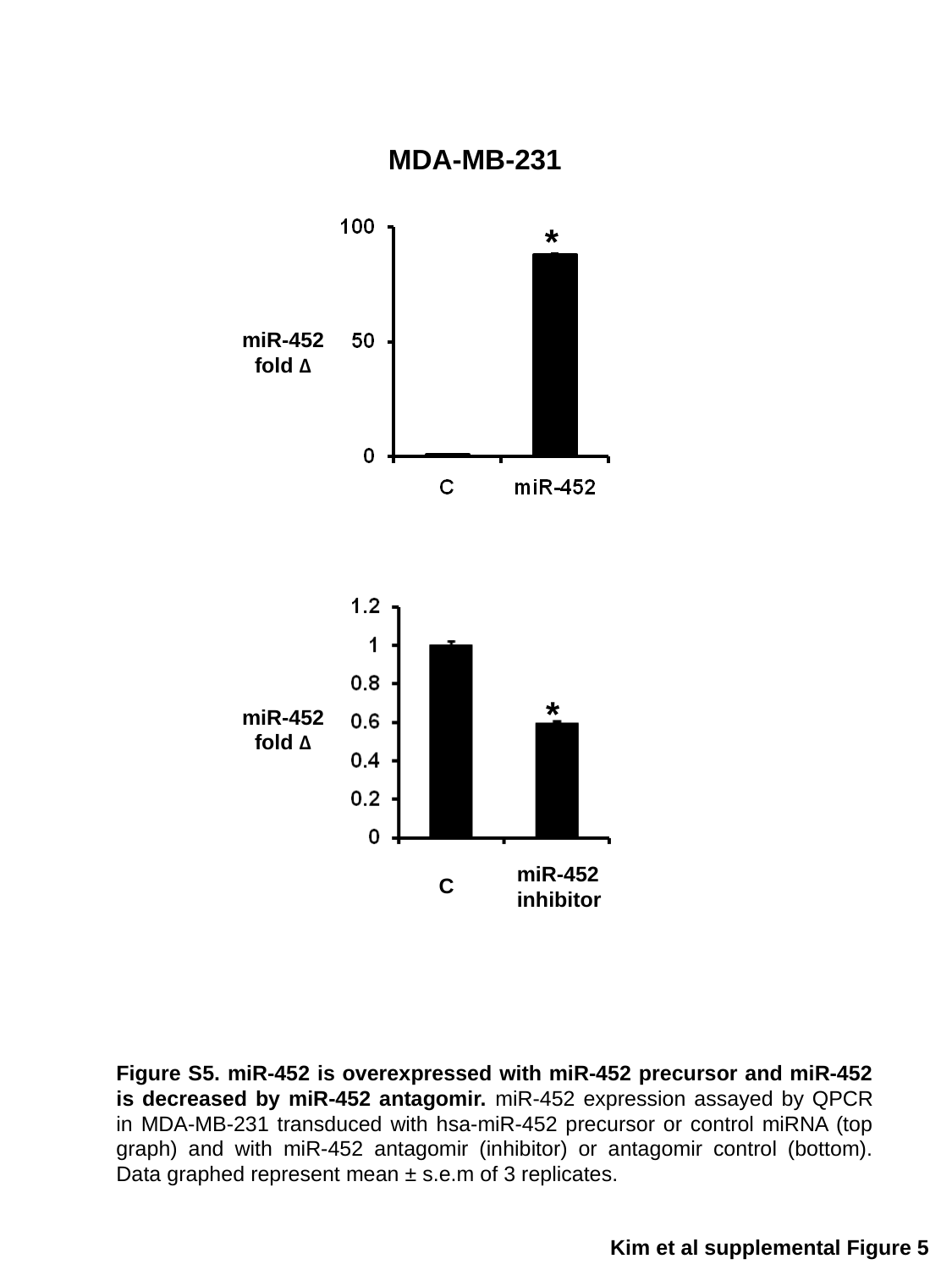

MDA-MB-231
*
 miR-452
 fold Δ
*
 miR-452
 fold Δ
miR-452 inhibitor
C
Figure S5. miR-452 is overexpressed with miR-452 precursor and miR-452 is decreased by miR-452 antagomir. miR-452 expression assayed by QPCR in MDA-MB-231 transduced with hsa-miR-452 precursor or control miRNA (top graph) and with miR-452 antagomir (inhibitor) or antagomir control (bottom). Data graphed represent mean ± s.e.m of 3 replicates.
Kim et al supplemental Figure 5

## Slide 6
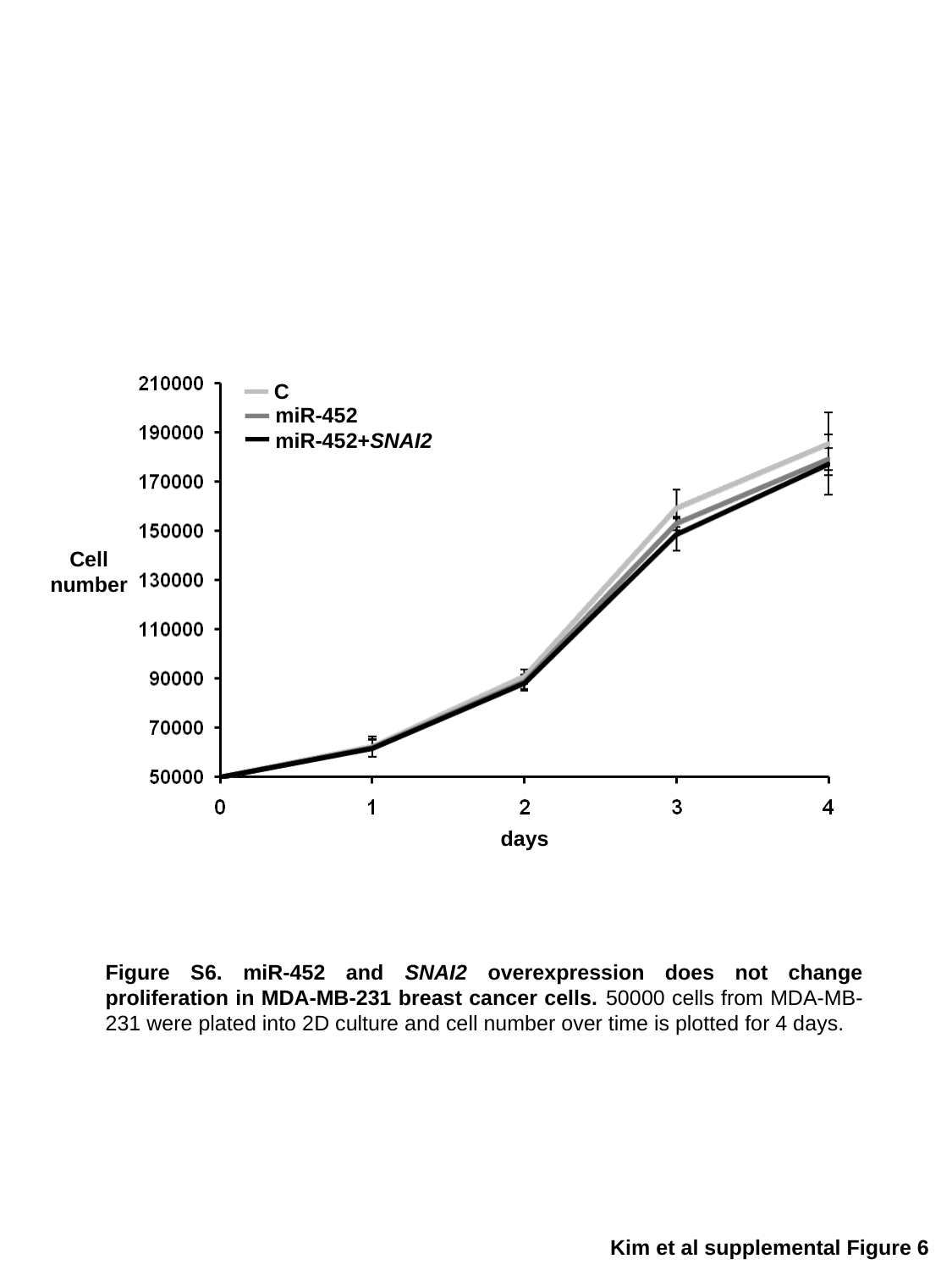

C
miR-452
miR-452+SNAI2
Cell number
days
Figure S6. miR-452 and SNAI2 overexpression does not change proliferation in MDA-MB-231 breast cancer cells. 50000 cells from MDA-MB-231 were plated into 2D culture and cell number over time is plotted for 4 days.
Kim et al supplemental Figure 6

## Slide 7
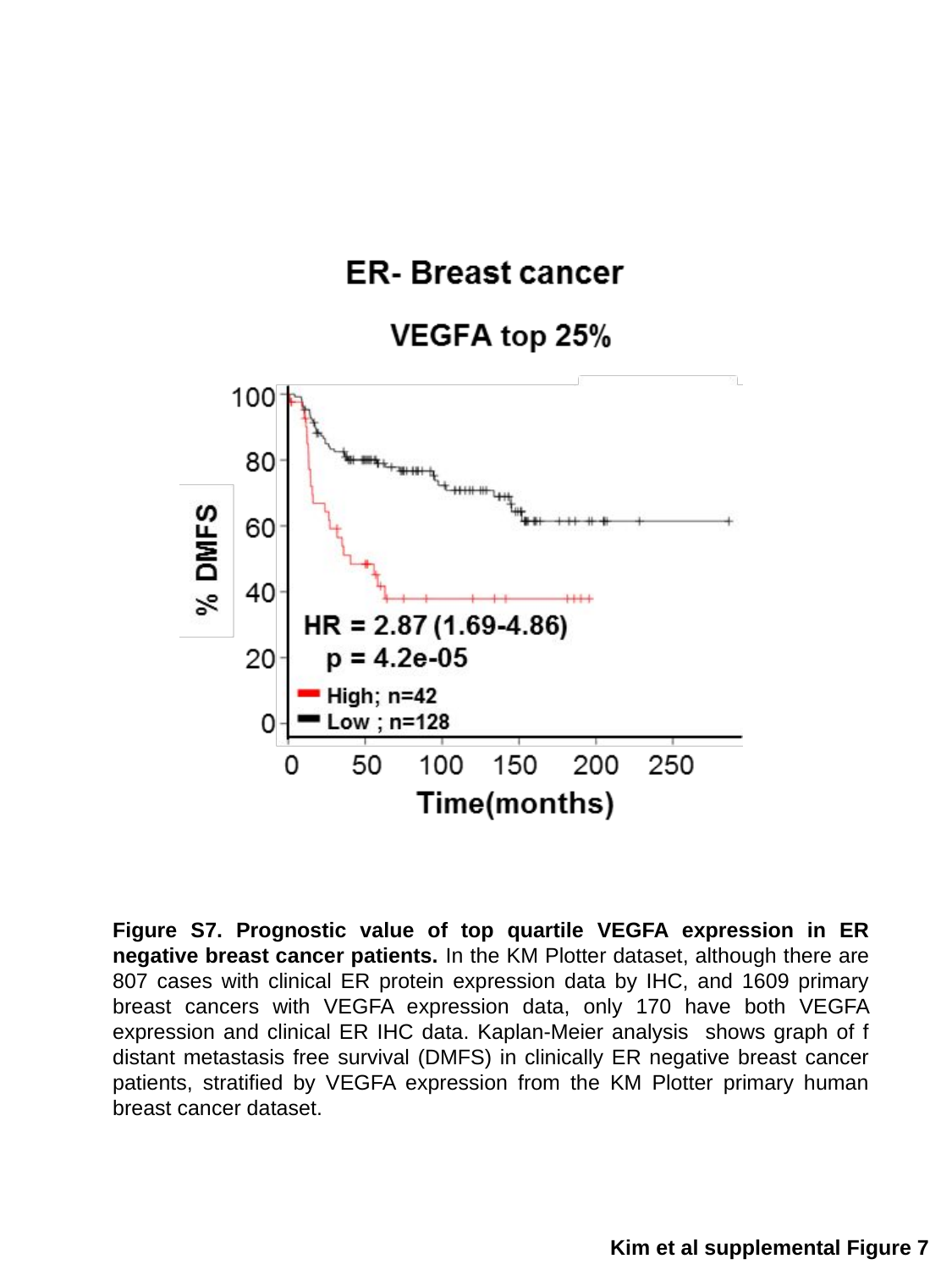

Figure S7. Prognostic value of top quartile VEGFA expression in ER negative breast cancer patients. In the KM Plotter dataset, although there are 807 cases with clinical ER protein expression data by IHC, and 1609 primary breast cancers with VEGFA expression data, only 170 have both VEGFA expression and clinical ER IHC data. Kaplan-Meier analysis shows graph of f distant metastasis free survival (DMFS) in clinically ER negative breast cancer patients, stratified by VEGFA expression from the KM Plotter primary human breast cancer dataset.
Kim et al supplemental Figure 7

## Slide 8
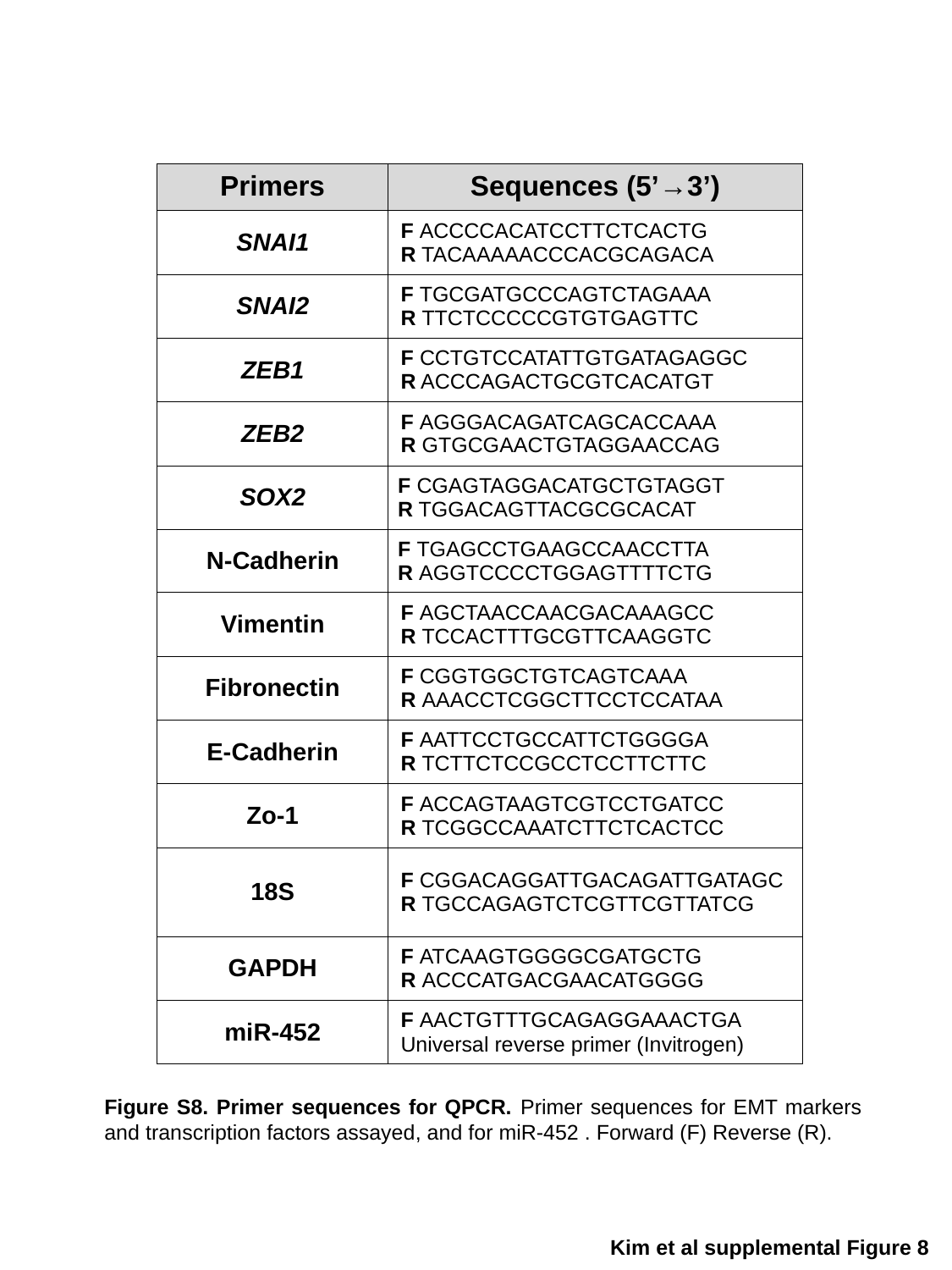

| Primers | Sequences (5’→3’) |
| --- | --- |
| SNAI1 | F ACCCCACATCCTTCTCACTG R TACAAAAACCCACGCAGACA |
| SNAI2 | F TGCGATGCCCAGTCTAGAAA R TTCTCCCCCGTGTGAGTTC |
| ZEB1 | F CCTGTCCATATTGTGATAGAGGC R ACCCAGACTGCGTCACATGT |
| ZEB2 | F AGGGACAGATCAGCACCAAA R GTGCGAACTGTAGGAACCAG |
| SOX2 | F CGAGTAGGACATGCTGTAGGT R TGGACAGTTACGCGCACAT |
| N-Cadherin | F TGAGCCTGAAGCCAACCTTA R AGGTCCCCTGGAGTTTTCTG |
| Vimentin | F AGCTAACCAACGACAAAGCC R TCCACTTTGCGTTCAAGGTC |
| Fibronectin | F CGGTGGCTGTCAGTCAAA R AAACCTCGGCTTCCTCCATAA |
| E-Cadherin | F AATTCCTGCCATTCTGGGGA R TCTTCTCCGCCTCCTTCTTC |
| Zo-1 | F ACCAGTAAGTCGTCCTGATCC R TCGGCCAAATCTTCTCACTCC |
| 18S | F CGGACAGGATTGACAGATTGATAGC R TGCCAGAGTCTCGTTCGTTATCG |
| GAPDH | F ATCAAGTGGGGCGATGCTG R ACCCATGACGAACATGGGG |
| miR-452 | F AACTGTTTGCAGAGGAAACTGA Universal reverse primer (Invitrogen) |
Figure S8. Primer sequences for QPCR. Primer sequences for EMT markers and transcription factors assayed, and for miR-452 . Forward (F) Reverse (R).
Kim et al supplemental Figure 8
